# Impact of Pesticides on Human Health in the Last Six Years in Brazil

**DOI:** 10.3390/ijerph19063198

**Published:** 2022-03-09

**Authors:** Monica Lopes-Ferreira, Adolfo Luis Almeida Maleski, Leticia Balan-Lima, Jefferson Thiago Gonçalves Bernardo, Lucas Marques Hipolito, Ana Carolina Seni-Silva, Joao Batista-Filho, Maria Alice Pimentel Falcao, Carla Lima

**Affiliations:** 1 Immunoregulation Unit of the Laboratory of Applied Toxinology (CeTICs/FAPESP), Butantan Institute, Vital Brazil Avenue, 1500, Butantan, São Paulo 05503-009, Brazil; adolfo.maleski@esib.butantan.gov.br (A.L.A.M.); leticia.lima@esib.butantan.gov.br (L.B.-L.); jefferson.bernardo@butantan.gov.br (J.T.G.B.); lucas-mh@hotmail.com (L.M.H.); anasenicarolina@gmail.com (A.C.S.-S.); jooaobsf@gmail.com (J.B.-F.); maria.falcao@esib.butantan.gov.br (M.A.P.F.); carla.lima@butantan.gov.br (C.L.); 2 Post-Graduation Program of Toxinology, Butantan Institute, São Paulo 05503-009, Brazil

**Keywords:** Brazilian scenario, pesticides, industrial agriculture, human health, toxic effects, rural workers

## Abstract

Every year, Brazil intensifies its activity in agriculture and, as a result, it has become one of the biggest consumers of pesticides in the world. The high rate of these substances raises environmental and human health concerns. Therefore, we collected papers from PubMed, Scopus, Scielo, and Web of Science databases, from 2015 to 2021. After a blind selection using the software Rayyan QCRI by two authors, 51 studies were included. Researchers from the South and the Southeast Brazilian regions contributed to most publications, from areas that concentrate agricultural commodity complexes. Among the pesticides described in the studies, insecticides, herbicides, and fungicides were the most frequent. The articles reported multiple toxic effects, particularly in rural workers. The results obtained can be used to direct policies to reduce the use of pesticides, and to protect the health of the population.

## 1. Introduction

Brazil is a country that economically relies on industrial agriculture for the production of a diverse range of soft commodities for exportation, with 88 million hectares cultivated in the country [1]. Agricultural production accounted for just over 5% of Brazil’s $1.8 trillion gross domestic product (GDP), with a variety of products from grain and oilseed chains, meats, sugar, biofuels, and fiber, to fruits and vegetables [2,3]. The harvested area has been expanding 23.48% per decade (1930 to 2017), while productivity has increased by 8% per decade, on average. This shows that the agricultural frontiers (agricultural and livestock activities) continue to expand in the country, mainly in the Amazon and Cerrado biomes [4].

Considering the increase in agricultural productivity in Brazil and the participation in the generation of GDP, the increase in agribusiness productivity has not been accompanied by a reduction in income inequality and poverty. Instead, strong alterations in the organization and use of the territory and the way of life of social groups in the affected biomes have been described [5]. In 2018, Brazil had 13.5 million people with per capita average earnings of 1.9 dollars (US$) per day, according to the criterion adopted by the World Bank to identify extreme poverty conditions. That figure is equivalent to the populations of Bolivia, Belgium, Cuba, Greece, and Portugal. Although the percentage of people in extreme poverty conditions has been stable compared to 2017, it increased from 5.8% in 2012 to 6.5% in 2018, a seven-year record [6].

Poverty can be aggravated by the productive agribusiness model that controls its food systems and its markets [7], as opposed to the choice of the food and nutrition security model that encompasses the realization of the right of everyone to permanently access quality food in sufficient quantity [8]. Consequently, the domestic consumer market may suffer supply shortages, favoring the export of food commodities. According to the Ministry of Development, Industry, and Foreign Trade, the devaluation of the Brazilian currency has been strongly increasing the competitiveness of Brazilian commodity exports to 2020, an increase of 79% year-on-year, and 9.8% more than 2019. From May to July alone, exports counted 701,061 tons. Meanwhile, the weak currency hindered imports, which totaled 373,557 tons in the period, down 12% year-on-year.

On the other hand, food such as rice, beans, bananas, and tomatoes that supply urban populations come, in large part, from family production, with huge potential for polyculture, organic, and agroecological farming. However, this sustainable agriculture fails to receive governmental support as incentives and subsidies [9,10] in contrast to the public policies of the European Union (EU), which aim for 2030 to halve the use of chemical pesticides and ensure that at least 25% of agricultural lands are set aside for organic farming, compared with the current 8% [11].

In order to fulfill the demand with a minimum loss, Brazilian commodity producers employ a large number of pesticides, estimated at 549,280 tons in 2018 [12,13]. According to an investigation by Unearthed, more than 1200 pesticides and herbicides, including 193 containing chemicals banned in the EU, have been registered in Brazil between 2016 and 2019. Almost half of all approved products contain active ingredients listed on the Pesticide Action Network’s list of highly hazardous pesticides, indicating that, in addition to serious environmental harm, they are related to toxic effects on human health [3,14,15].

Based on scientific evidence, the real risks that pesticides pose to human health (occupational and consumer exposure) and the environment are fully justified [16,17]. They cause health conditions from acute reactions in the skin and respiratory system to chronic diseases including hematologic and hormonal abnormalities, infertility, miscarriages, fetal malformation, neurological diseases, and cancer. The underlying mechanisms of these effects are genotoxic, neurotoxic, and endocrine-disrupting actions [18,19,20].

The worsening of poverty and the loss of guarantee of food security are examples of the negative socio-economic impact of the massive use of pesticides in Brazil. Of equal importance are its direct effects on the environment, non-target organisms, and human health [21,22]. A substantial task must be carried out by state agencies, non-governmental organizations, and researchers from universities and institutes to create scientific documentation that guides public policies in the adoption of the highest standards of procedures, guidelines, and mitigation measures to reduce potential risks to the population [23], as well as initiatives capable of circumventing its social and environmental damage. Therefore, this work aims to understand the scenario of Brazilian research that portrays the various toxic effects of pesticides on human health carried out in the last six years.

## 2. Methods

### 2.1. Data Sources

**Figure 1:**
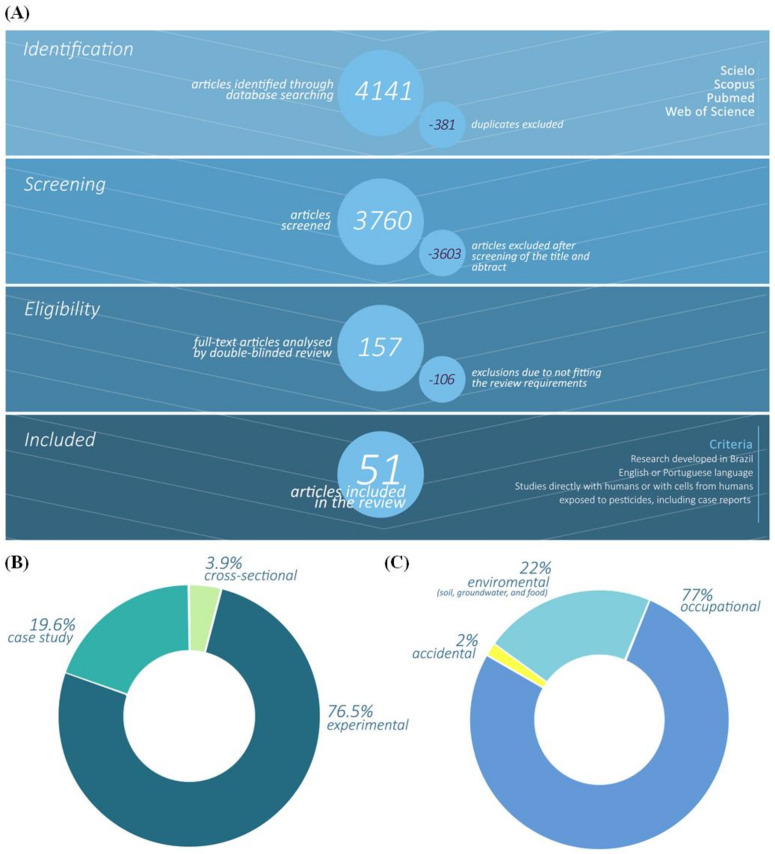
(**A**) Study Flow of selected articles. (**B**) Type of the studies conducted about pesticides in Brazil between 2015 and March 2021 (8th of March). (**C**) The route of exposure to pesticides were grouped into three main categories: occupational, environmental, and accidental, as described by the authors of the articles included in this review.

Online searches of the published literature within the last six years, 2015 to 2021, were conducted through the databases SCIELO, SCOPUS, PUBMED, and WEB OF SCIENCE. On the 8th of March of 2021, we utilized the strategy of search in all these databases using the terms through the keywords pesticides, humans, and Brazil: ((“pesticides”(MeSH Terms) OR “pesticides”(All Fields)) OR (“pesticides”(Pharmacological Action) OR “pesticides”(MeSH Terms) OR “pesticides”(All Fields))) AND (“humans”(MeSH Terms) OR “humans”(All Fields) OR “human”(All Fields)) AND (“brazil”(MeSH Terms) OR “brazil”(All Fields)) AND (“2015/01/01”(PDAT): “3000/12/31”(PDAT)). All searches together resulted in 4141 articles that, after evaluation and selection by members of the research team, were restricted to 51. A full description of the search strategy is provided in Figure 1A.

### 2.2. Studies Selection

Using the online software Rayyan (http://rayyan.qcri.org/, accessed on 27 January 2022), 381 article duplications were excluded. Two reviewers carried out a double-blind review and independently screened paper titles, index terms, and abstracts to identify relevant articles for possible inclusion. The discrepancies were resolved by a third reviewer. It was used as the first inclusion criteria research developed in Brazil involving pesticides and articles written in English or Portuguese. Overall, 3603 articles that did not accomplish these criteria were excluded.

Next, a second round of more solid review was performed with the included articles where each work was independently read by two different reviewers using a second set of inclusion criteria, i.e., research developed in Brazil involving pesticides, articles in English or Portuguese, and research involving direct studies in humans or human cells exposed to pesticides, including case reports. The articles that did not fit these criteria (106) were excluded. Thereby, 51 works were selected and evaluated concerning the Brazilian institutions that published studies about human exposure to pesticides, the number of articles published per year, the type of study conducted, the regions where the studies were conducted, the variety of crops that humans had contact with, the type of pesticides exposure evidenced, the effects observed in the exposed populations, the chemical group, function, and the distribution per region of the different pesticides mainly applied.

## 3. Results and Discussion

The Brazilian population has been exposed to pesticides used in the production of commodities directly through dermal, oral, eye perfusion, and respiratory airways (notably rural workers), as well as indirectly through residues consumption in food and water [22,24,25,26,27]. The Brazilian Association of Collective Health [28] estimates that pesticides contaminate approximately 70% of food consumed by Brazilians, and they drink nearly 7.5 L of pesticides per year—the highest per capita consumption rate in the world.

Another important route of contamination is domestic or occupational exposure to multiple pesticides during pregnancy, which determines changes in fetal development and serious complications during childhood [29]. Mavoungou et al. [30], using data from the two French national population-based case-control studies, ESCALE (2003–2004) and ESTELLE (2010–2011), demonstrated a positive correlation between domestic and occupational exposures to pesticides during pregnancy with both childhood non-Hodgkin and Hodgkin lymphoma. Moreover, contaminated breast milk leads to pronounced immunological deficiencies in the newborn, increasing the risks of infections, mainly meningitis and inner ear infections in infants [31,32].

In 2017, the National Toxic-Pharmacological Information System (SINITOX) reported 2548 cases of pesticides contamination in Brazil [33]. Taking into account that pesticide intoxications are not considered a problem of compulsory notification in Brazil (according to Ordinance No. 777/GM, 28/04/2014) and that the Ministry of Health itself estimates that for each notified pesticide intoxication event, there are another 50 unnotified [34,35,36], human cases of pesticide intoxication is an alarming and neglected health problem in Brazil.

In this context, initiatives have been conducted by groups in some Brazilian research institutions and governmental and non-governmental organizations to minimize the problem of deficient notification. Other efforts include expanding the identification of sociodemographic conditions, the use of personal protective equipment, history of poisoning and hospitalizations for pesticides, and the existence of primary health care units for rural workers [37,38,39,40,41,42].

In this work, our purpose was to gather information about studies conducted by Brazilian research groups over the effects of pesticides on human health in the last six years. We revised case studies, and cross-sectional and experimental data (Figure 1B) of reported intoxications in humans derived mainly from occupational (77%) and environmental (21.3%) repeated exposure to pesticides (Figure 1C).

**Figure 2:**
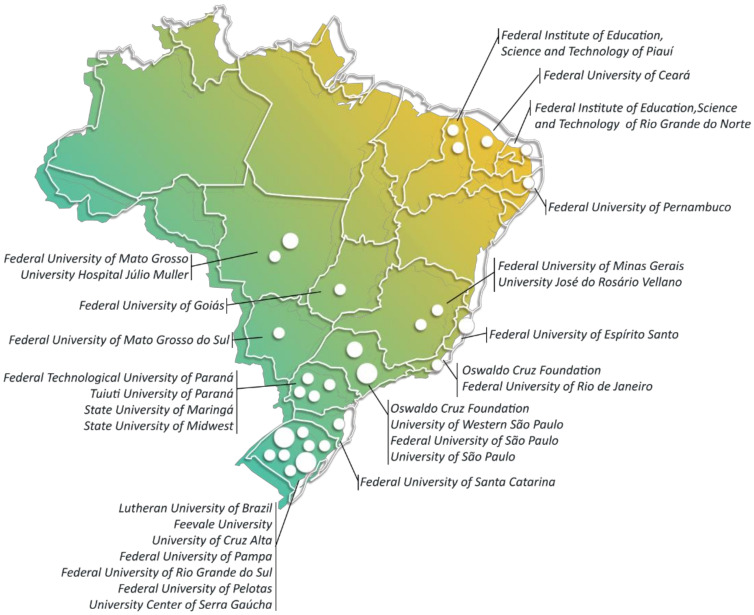
Brazilian institutions that published studies about human exposure to pesticides from 2015 to March 2021, classified by regions. The 51 articles included in the systematic review were grouped according to the research institutions that carried the study.

We found that among the fifty-one articles included in this systematic review, researchers from four of the five Brazilian administrative regions contributed to all publications, with 23 (46.2%) articles from institutions of the South region and 16 (30.7%) from the Southeast region, and seven and five (13.5% and 9.6%) articles generated by institutions from the Northeast and Midwest regions, respectively (Figure 2).

**Figure 3:**
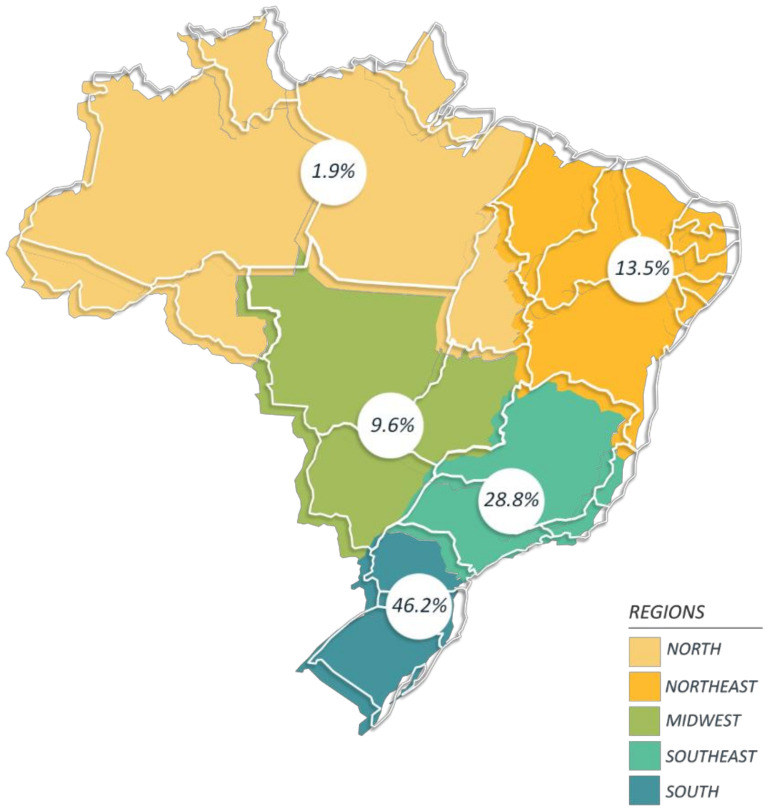
Percentage of Brazilian geographical regions where the studies were conducted, from 2015 to March 2021. The 51 articles included in the systematic review were grouped according to the Brazilian regions where the research was conducted.

Although articles developed by researchers from institutions of the North region were not included, Freire, Koifman, and Koifman [43] from the National School of Public Health at Rio de Janeiro detected the presence of 24 types of organochlorine pesticides in the plasma of 978 adults exposed to different pesticides in Rio Branco, the capital of Acre (Figure 3). The results of this study highlight the positive association between high levels of pesticides (beta-HCH, p,p′-DDE-1,1-dichloro-2,2-bis(p-chlorophenyl) ethylene, and hexachlorobenzene) in the serum of male adults and alterations in hematological parameters such as eosinophilia, low hemoglobin content, and erythropenia, and high levels of liver metabolism enzymes such as bilirubin, glutamic-oxaloacetic transaminase, and glutamic-pyruvic transaminase.

The Amazon region in the states of Acre, Amapá, Rondonia, Tocantins, and Amazonas has been suffering from deforestation due to many official policies [44,45], with large natural areas replaced by monoculture with an indiscriminate spread of pesticides. Soy cultivation is a major driver of deforestation in the Amazon basin. Seeds from the genetically modified (GM) soybean plant provide high protein animal feed for livestock, and 80% of Amazon soy is destined for animal feed; smaller percentages are used for oil or consumption [46]. The growing use of the land for monoculture and the consequent application of pesticides calls for a new direction in research purposes carried out by institutions of this region. Investigations are required to understand the impact of the growing and indiscriminate use of pesticides on the health of the Amazon population directly associated with exposure to pesticides or the use of contaminated water since the region is home to one of the largest hydrographic basins in the world.

Both surface water and groundwater pollution caused by pesticides are very serious and cause urgent issues in freshwater and coastal ecosystems worldwide [47]. Such pesticide contamination in water not only directly impacts the drinking water quality in local areas, but also causes indirect impacts by transferring across species, such as in soil and the food chain. According to the Organization for Economic Co-operation and Development (OECD) [48], agriculture in the EU contributes 40–80% of total nitrogen and 20–40% of phosphorus to the pollution of surface waters. The United States Geological Survey (USGS) found several pesticides in more than 90% of water and fish samples collected from United States (US) streams [49]. Brazilian studies indicate that pesticide contamination had been reported in surface water, in levels exceeding the national standard, varying in the different seasons.

de Castro Lima et al. [50] confirmed the presence of high levels of four herbicides (2,4-D, atrazine, deethyl-atrazine, and simazine), three fungicides (carbendazim, tebuconazole, and epoxiconazole), and one insecticide (imidacloprid) in the water of Guaporé River, in the Rio Grande do Sul and its tributaries, that irrigate soybean, corn, and winter cereals crops and grassland forage production region. In addition, water samples collected in the river banks in the area of an agricultural project in Formoso do Araguaia city, Tocantins demonstrated levels of substances with potential for groundwater contamination such as clomazone (0.376 µg·L^−1^), fluazifop-p-butyl (<0.020 µg·L^−1^), flutolanil (<0.020 µg·L^−1^), metsulfuron-methyl (<0.020 µg·L^−1^), propanil (<0.006 µg·L^−1^), and imidacloprid (0.065 µg·L^−1^) [51].

These measures, added to the analysis of pesticide residues in human tissues and samples corroborate the guidelines stipulated by the OECD (Pesticide Assessment and Testing Project, 2013) [48], which recommend the application of a systematic assessment of environmental and social risks. The dosage of pesticides or their metabolites in human samples, such as hair and blood collected from workers in the South and Southeast regions of Brazil, has been evaluated. The results demonstrated the presence of arsenic (3.776 µg·L^−1^), nickel (2.686 µg·L^−1^), manganese (2.048 µg·L^−1^), zinc (1.442 µg·L^−1^), and cooper (1.939 µg·L^−1^) as a predictive risk factor for the development of disorders associated with chronic exposure to pesticides [52,53,54]. Moreover, monitoring systems to identify the spatial distribution of the planted area of crops, consumption of pesticides, and health problems related to chronic occupational exposition in Brazil have been carried out [55,56,57,58,59,60,61] with the purpose to integrate data on exposure to pesticides to social, economic, and environmental conditions, and to identify the bottlenecks in the control of the indiscriminate use of various pesticides. The data from these articles confirmed a positive correlation between the use of million liters of pesticides sprayed on soybean, corn, and sugarcane crops with the development of health problems in Mato Grosso, Paraná, and Rio Grande Sul, areas that concentrate agricultural commodity complexes.

As demonstrated in the review, these data corroborate the previous survey performed between 2012 and 2014 by Bombardi [62] that showed Mato Grosso, Mato Grosso do Sul, Goiás, and São Paulo as the significant consumers of pesticides in Brazil (44–92% more than the national average). Since then, according to the Brazilian Ministry of Agriculture [3,23], records of new pesticides approved in 2019 are the highest in the historical data series. The average approval of new pesticides has been more than one per day. This is indicative that with access to new types and formulations [63,64], the number of tons of pesticides sprayed in the conventional or GM crops will be intensified, reinforcing the establishment of policies of continuous monitoring of pesticides in the environment by government agencies to maintain an updated inventory on the effects of contamination over non-target organisms and the environment.

**Figure 4:**
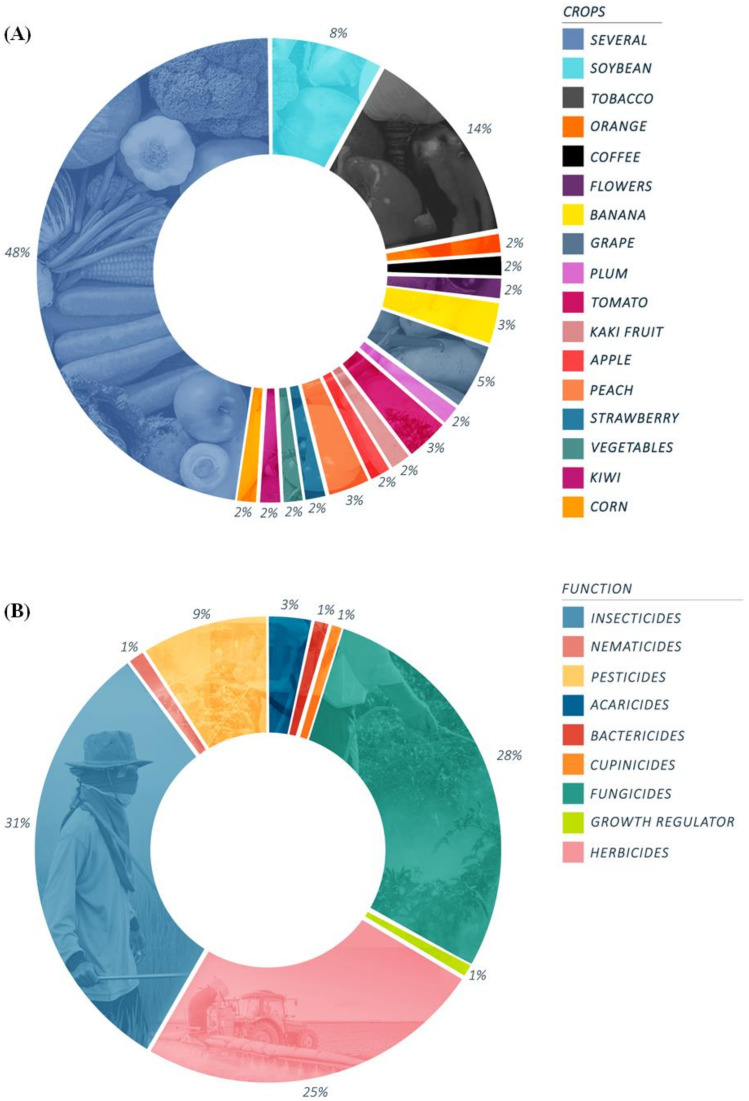
Percentage of the different crops and function of pesticides described in the articles between 2015 and March 2021. The studies included in the systematic review were grouped according to (**A**) the type of crops that humans had contact with. The majority of the studies (47.6%) include different types of plantations such as vegetables and cereals, a classic sign of polyculture activity present in Brazil. (**B**) Function described by the authors or the pesticide package leaflet. Some of the chemicals mentioned have more than one function described; therefore, they were included in different categories.

**Figure 5:**
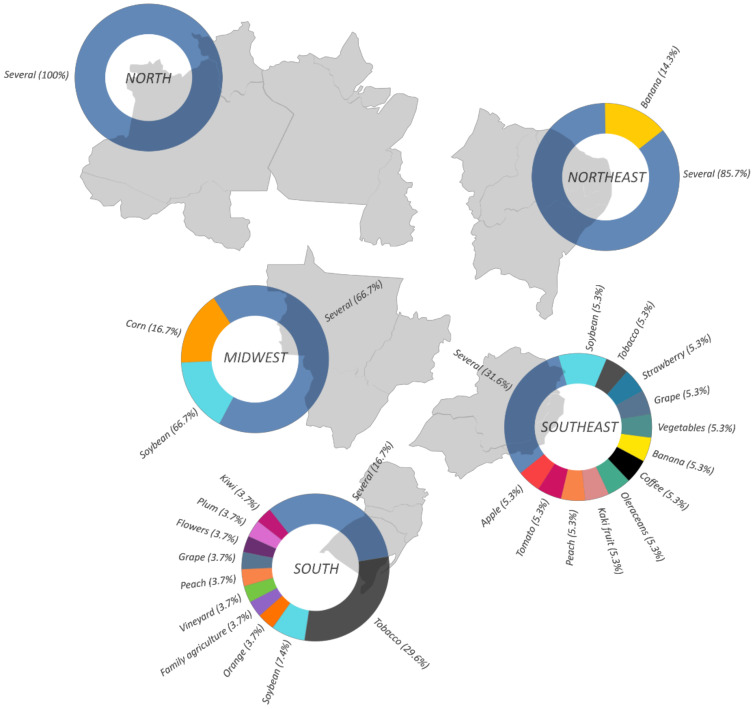
Geographic distribution by percentage of the different crops described in the articles between 2015 and March 2021. The 51 studies included in the systematic review were grouped according to the type of crops and distributed according to the region’s occurrence in the percentage of total crops. The southern and southeastern regions of Brazil have a greater variety of monoculture plantations, while in other regions, polyculture stands out.

Then, we analyzed the correlation between the toxic effects described in the articles reviewed with the most prevalent crops in Brazilian regions. Our data show a direct relationship with the agricultural production in each Brazilian region. Figure 4A shows that 14.3% of the articles reported the use of pesticides in the cultivation of tobacco, and 7.9% and 4.8% each in the cultivation of soy and grape, followed by 3.2% of articles related to the use of pesticides in banana, tomato, and peach crops. Crops such as kiwi, plum, apple, coffee, orange, flowers, persimmon, strawberry, and other vegetables corresponded to 19% of the articles. Moreover, 47.6% of the articles report the use of pesticides in various crops without specifically naming them, but it can be interpreted based on the crop profile of each region. In Figure 5, we observed an overlapping of data with the different cultures in each region in Brazil.

In 2019, the Midwest and Southeast regions of Brazil equally reached the highest value of agricultural commodity production (30%), followed by the South region with 28% and the North and Northeast regions with equal production of 6% [65]. In all major regions, the main product was soy, except for the Southeast region, which has sugar cane as the highest crop produced. Further, soybean (34.8%), sugar cane (15.2%), corn (13.2%), coffee (4.9%), and cotton (4.4%) represent, in this order, the commodities most produced by Brazilian regions. Taken together, these data show the amplitude of pesticide contamination to agribusiness workers as well as familiar agriculture in these regions since those are very pesticide-demanding practices [37,38,42,66].

Pesticides are classified according to the type of activity/target organism and to the chemical nature as insecticides (chlorinated hydrocarbons, organophosphates, carbamates–insecticides, and pyrethroids); fungicides and bactericides (dithiocarbamates, benzimidazoles, triazoles diazoles, and diazines morpholines); and herbicides (phenoxy hormone products to control growth and division like triazines, amides, carbamates-herbicides, dinitroanilines, urea derivatives, sulfonyl urea, bipyridyls, and uracil) among others [13].

Analyzing the type of pesticides, we found that most articles in this review describe toxic effects related to exposure to insecticides (31%), followed by fungicides (28%), herbicides (25%), and pesticides (generic name, 9%). Acaricides represented 3% of the articles, and 4% describe the toxic effects induced by nematicides, bactericides, cupinicides, and growth regulators together (Figure 4B). Our data corroborate the findings that show organophosphate Glyphosate-based herbicides as the world’s leading post-emergent, broad-spectrum, and non-selective herbicides for the control of annual and perennial weeds [67,68,69].

Glyphosate is the most sold active ingredient in Brazil, with 195,056 tons commercialized in 2018 [70]. It was recently reclassified by ANVISA (Brazilian Health Surveillance Agency, 2018) as class III, hazardous for the environment. In Brazil, the 65 µg·L^−1^ maximum limit concentration in superficial waters was determined by the National Council of Environment- Conama by resolutions #357/2005 and #20/1986. According to the Brazilian Ministry of Health Ordinance #518/2004, the maximum permissible concentration of Glyphosate in drinking water destined for human consumption is 500 µg·L^−1^ [71], similar to the high concentrations set by Environmental Protection Agencies in the United States [72], European Union [73], and Australia [74], where limits for drinking water are 700 µg·L^−1^, 0.1 µg·L^−1^, and 1000 µg·L^−1^, respectively.

This problem has been reported in Brazil [75,76]. de Castro Lima et al. [50] shows that the use of pesticides in rural catchments leads to the contamination of surrounding water resources. They described that 17 out of 18 water samples from the South region were contaminated with at least one pesticide (atrazine, simazine, propoxur, imidacloprid, carbendazim, azoxystrobin, thiamethoxam, fipronil, propiconazole, tebuconazole, and carbofuran).

Unfortunately, the establishment of high concentration limits in water for human consumption to substances with a good toxicological understanding by the environmental protection agencies in the main American countries (Brazil, United States, and Canada) does not follow the precautionary principle. On the contrary, in the EU, environmental regulation is required to fulfill the principles established in Article 174 of the EU Treaty, so it offers a high level of protection and is consistent with the precautionary principle. Pesticides in drinking water are regulated in the EU by the Drinking Water Directive (Directive 98/83/EC), with value of 2.5 µg·L^−1^ for Bisphenol-A, as benchmark. The parametric values laid down in this Directive are based on the scientific knowledge available and the precautionary principle, and are selected to ensure that water intended for human consumption can be consumed safely on a life-long basis, thus ensuring a high level of health protection.

Numerous in vivo studies (reviewed by Disner et al. [77]) have recursively proven that exposure to pesticides, either isolated or in combination [78], affect human health due to their actions as carcinogens [79,80,81,82,83,84], neurotoxicants [85,86,87,88], endocrine disruptors, developmental toxicants [89,90,91,92], and metabolic toxicants [93,94].

**Figure 6:**
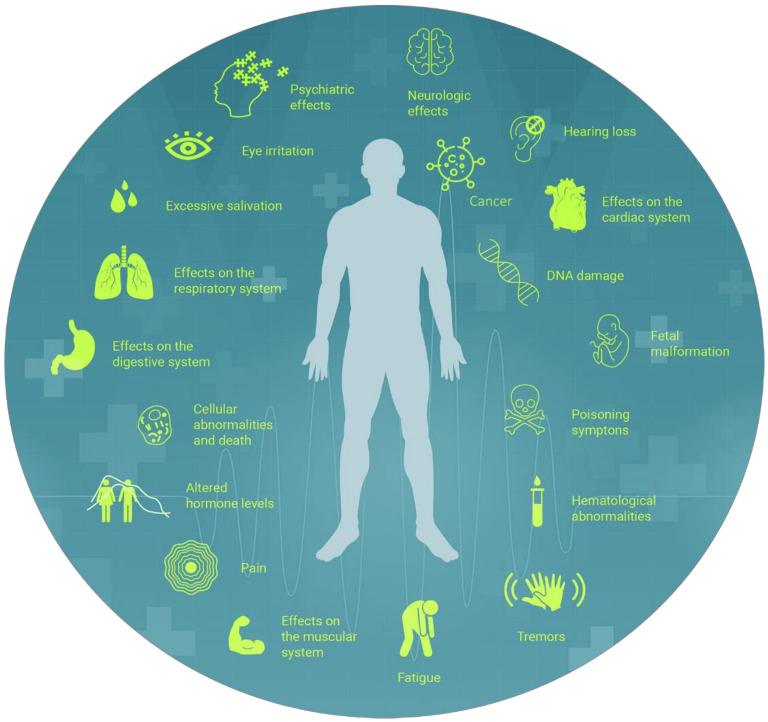
The repertoire of effects observed in the studied populations exposed to pesticides. Illustration of the types of harmful effects on humans involved in the studies caused by the exposure to different types of pesticide mentioned in the articles. All the effects were cited at least once, with most of the effects being associated and mentioned in different studies.

The impact on human health of environmental exposures is a challenge due the variability in time and space, which makes it difficult to delineate their potential harmful on the cellular, organ, and organism level. The articles selected here reported multiple toxic effects of pesticides, particularly inflicting rural workers, inducing from hematological abnormalities, DNA damage, and cell death to excessive salivation, skin and eye irritations, pain, altered hormone levels, infertility, miscarriages and fetal malformation, neurological symptoms, such as tremors and fatigue, hearing loss, psychiatric effects and suicides, neurodegenerative diseases, effects on muscular and cardiac systems, development of related metabolic diseases, including overweight, underweight, insulin resistance and even diabetes, and various types of cancer (Figure 6).

The studies revealed that the most evident effect was genotoxicity, altering the metabolic and oxidative pathways and provoking DNA damage and epigenetic changes. Moreover, the intricacy of pesticide’s metabolic characteristic is augmented by co-exposition to other intoxicants that increased or decreased enzymes implicated in metabolism [84,95,96,97,98,99,100,101,102,103,104,105] However, it is noteworthy that, despite these findings, environmental exposure is not an isolated factor for these diseases. It can act as a catalyst or summative factor to pre-existing conditions such as: unhealthy diet, sedentary lifestyle, tobacco smoking, and alcohol drinking.

According to Smith et al. [106], carcinogens generally exhibit more than 1 of the 10 main characteristics, such as genotoxicity, alteration of DNA repair systems or genomic instability, operate as electrophiles directly with or after metabolic activation, cause oxidative stress and chronic inflammation, immunosuppression, control of actions mediated by the receptor, induce immortalization or modify cell growth, induce cell death, or block the supply of nutrients and cause epigenetic changes [56,80,82,83,107,108,109]. Therefore, DNA damage and epigenetic alterations caused by chronic exposure to multiple pesticides are directly related to the development of several diseases, including different types of cancer.

Pesticides may cause a transient or permanent alteration of the immune system, leading to higher risks for chronic health disorders, including hematological and immune alterations such as inflammation and cytokine modulation [98,110,111,112,113]. Recently, the intestinal microbiota has emerged as a notable factor regulating pesticides’ toxicity. Giambò et al. [114] propose that pesticides can disrupt the typical composition and functionality of the gut microbiome, leading to significant metabolic imbalances, especially in glycolipid metabolism. On the other hand, the bacterial community responds to pesticide toxicity by promoting the growth of bacterial strains most involved in the detoxification mechanisms of these chemical compounds.

A very interesting view is that the epithelial barrier integrity in the airways, gut, and esophagus, essential for homeostasis control, can be affected by multiple environmental toxic agents, such as pesticides. Akdis [115] proposes that the increase in agents that damage the epithelial barrier underlies not only the development of allergy and autoimmune conditions in barrier-damaged tissues but also a wide range of diseases in which an immune response to commensal bacteria and opportunistic pathogens occurs. The development of permeable epithelial barriers leads to microbial dysbiosis and bacterial translocation to interepithelial and subepithelial areas, and the development of tissue microinflammation. Notably, the microbial-mediated effects potentially modulate the course of the neurological disorders which display a chronic state of inflammation in the periphery as well as in the brain [116], providing an opportunity to intensify public policies in adopting the highest standards of measures to reduce the risks of pesticides for the population.

Data from hematological tests of rural workers from the Southeast and South Brazilian regions exposed to organochlorines (OCs) and dithiocarbamate pesticides show hematological alterations, such as neutrophilia [117] or leucopenia [118,119]. The impact of chronic exposure to multiple pesticides was described in the immune response, showing in the plasma of exposed farmers compared to controls increased levels of the pro-inflammatory cytokines such IL-6 [105] and IL-1β and TNF-α [120], and augmented levels of C3, a key component in complement activation, amplification, and effector generation [119].

Pesticides may adversely affect hematopoietic tissue and liver functions in populations chronically exposed to high levels of these compounds [121]. Some experimental studies by Brazilian groups corroborate the evidence for pesticide hepatotoxicity, disrupting biochemical parameters, and antioxidant capacity. Soybean farmers in southern Brazil during high pesticide exposure periods presented lower butyrylcholinesterase (BChE), increased aspartate aminotransferase (AST), and ferric reducing ability of plasma (FRAP) activities, as well as high levels of urea and creatinine in the blood [122,123].

Lermen et al. [124] described the hepatotoxic effect in farmers who grow citrus in the Vale do Caí, in the Rio Grande do Sul, southern Brazil. Freire, Koifman, and Koifman [43], besides observing eosinophilia, low hemoglobin levels, and low erythrocyte count among residents in an area heavily contaminated with OCs, detected high levels of bilirubin, glutamic-oxaloacetic transaminase (GOT), glutamic-pyruvic transaminase (GPT), and gamma-glutamyl transferase (GGT). Moreover, alterations of renal functions were evidenced in children from a tobacco-producing region [66,125].

Endocrine-disrupting chemicals (EDCs) comprise a group of compounds that have been examined extensively due to the potential detrimental effects on human health. Animal and in vitro studies support the conclusion that endocrine-disruptors affect the hormone-dependent pathways responsible for male and female gonadal development [126], either through direct interaction with hormone receptors or via epigenetic and cell-cycle regulatory modes of action. In humans, most studies point to an association between exposure to EDCs and male or female reproduction systems disorders, such as infertility, endometriosis, breast cancer, testicular cancer, or low quality or dysfunctional sperm [89,90,91,92].

The OCs and organophosphorus (OPs) are examples of pesticides with endocrine-disrupting properties. Moreover, the toxic effects they induce on the human reproductive system are directly related to the dose, the frequency of exposure, the route of exposure, and the genotypic characteristics of the affected populations [127]. Chronic exposure to OPs can be monitored by assessing plasma cholinesterase identified as a marker [122,124,125,128]. The occupational exposure of adults to pesticides such as parathion and methyl parathion increases the risk of morphological abnormalities in the sperm, including a decline in sperm count, a decreased percentage of viable sperms, and a reduction in the seminal volume [129,130].

The impact of pesticides on human thyroid functions, essential in the growth and development of children and adolescents, weight, memory, regulation of menstrual cycles, fertility, concentration, mood, and emotional control was recently investigated in soybean farmers in southern Brazil. Low levels of thyroid-stimulating hormone (TSH) and increased levels of total triiodothyronine (TT3) and free thyroxine (FT4) were detected by Bernieri et al. [122]. Furthermore, Cremonese et al. [131] described altered sperm morphology, high sperm count, and low luteinizing hormone (LH) and prolactin levels in young rural men with poorer backgrounds relative to urban subjects. Santos et al. [132] found positive associations of lifetime years of agricultural work with reduced total thyroxine (T4) and increased male testosterone; and of lifetime agricultural work and use of various pesticide classes (i.e., insecticides, herbicides, organophosphate insecticides, dithiocarbamate fungicides, and pyrethroids), mancozeb (fungicide), and paraquat.

Interestingly, prenatal exposure to OPs in humans has a greater impact on fetal growth and development in early childhood [133]. Prenatal indoor exposure to pesticides (e.g., chlorpyrifos, OPs, and vinclozolin) and herbicides (such as triazines and metolachlor) has been suggested to increase teratogenicity risk [29] due to the high susceptibility of most fetal systems during certain periods of development [134]. Robust data show a positive association between maternal pesticide exposure during the three months that preceded conception and the first three months of pregnancy, and paternal pesticide exposure during the 12 months that preceded conception and the occurrence of congenital malformations in children in Mato Grosso [135].

Agricultural workers who have concurrent exposure to pesticides are at increased risk of hearing loss in low and high frequencies [136,137,138]. Tobacco farmers from southern Brazil exposed to pesticides exhibited signs of central auditory dysfunction characterized by decrements in temporal processing and binaural integration processes/abilities [139,140]. Using meatoscopy, pure tone audiometry, logoaudiometry, high-frequency thresholds, and immittance testing, Tomiazzi et al. [141] demonstrate the direct effect of pesticides on hearing loss in 127 participants, of both sexes, aged between 18 and 39, carried out in Pontal do Paranapanema region, one of the less developed regions of the state of São Paulo.

The nervous system is particularly susceptible to many pesticides of several distinct chemical classes. Several studies show that prenatal and early childhood exposure to OPs is associated with neurodevelopmental effects [142] and neurocognitive disorders as attention deficit disorder with or without hyperactivity (ADHD) and autism spectrum disorder (ASD) [143]. A meta-analysis concluded that low-dose exposures to OPs were linked to reduced psychomotor speed, executive function, and visuospatial ability, as well as work and visual memory [144]. Other studies have also associated OCs, OPs, and other pesticides with dementias such as Alzheimer’s disease and amyotrophic lateral sclerosis, but mainly with Parkinson’s disease [145,146]. Campos et al. (2015) [147] demonstrated that children and adolescents living in Cidade dos Meninos in the Brazilian State of Rio de Janeiro contaminated with OC pesticides presented cognitive deficiencies. In the same state, a major impact of pesticide exposure in the development of tremor was observed [148].

Hazardous pesticides (classes Ia, Ib, and II), such as the OP insecticides monocrotophos, phorate, and methyl parathion or the herbicide paraquat [149], have been responsible for most pesticide suicides worldwide over the last five decades. Several other countries where pesticide suicide is a significant problem have reported the effects of national pesticide regulation on suicide [150], notably Bangladesh [151], South Korea [152], Sri Lanka [153], and India [86]. Although regulatory agencies have many options for increased safeguards for any pesticide, the most effective and reliable is to ban the most dangerous pesticides and those with higher potential for harm to humans and the environment following the precautionary principle. Finally, more severe cases of mental illness, such as depression and attempted suicide, have also been reported by groups in some Brazilian research institutions [154,155,156], which leads us to advocate for the importance of strengthening the evaluation of mental illness caused by chronic exposure to pesticides, especially in rural workers living in low economic and social conditions by health authorities.

## 4. Conclusions

The review demonstrated that over the last six years, important Brazilian institutions have been dedicated to studying the possible effects of pesticides on human health. An important factor, since Brazil is an agricultural country, is that pesticide use increases every year. Furthermore, scientific publications on the effect of pesticides on human health play a fundamental role in guiding public policies in the adoption of the highest standards of procedures, guidelines, and mitigation measures to reduce potential risks to the population.

## Data Availability

The original contributions presented in the study are included in the article, further inquiries can be directed to the corresponding author.
